# Chronic Inflammation: A Common Promoter in Tertiary Lymphoid Organ Neogenesis

**DOI:** 10.3389/fimmu.2019.02938

**Published:** 2019-12-18

**Authors:** Shanshan Luo, Rui Zhu, Ting Yu, Heng Fan, Yu Hu, Sarajo Kumar Mohanta, Desheng Hu

**Affiliations:** ^1^Institute of Hematology, Union Hospital, Tongji Medical College, Huazhong University of Science and Technology, Wuhan, China; ^2^Department of Integrated Traditional Chinese and Western Medicine, Union Hospital, Tongji Medical College, Huazhong University of Science and Technology, Wuhan, China; ^3^Institute for Cardiovascular Prevention, Ludwig-Maximilians-University, Munich, Germany

**Keywords:** immunity, inflammation, atherosclerosis, tertiary lymphoid organs, adventitia

## Abstract

Tertiary lymphoid organs (TLOs) frequently develop locally in adults in response to non-resolving inflammation. Chronic inflammation leads to the differentiation of stromal fibroblast cells toward lymphoid tissue organizer-like cells, which interact with lymphotoxin $\alpha_{1}\beta_{2}^{+}$ immune cells. The interaction initiates lymphoid neogenesis by recruiting immune cells to the site of inflammation and ultimately leads to the formation of TLOs. Mature TLOs harbor a segregated T-cell zone, B-cell follicles with an activated germinal center, follicular dendritic cells, and high endothelial venules, which architecturally resemble those in secondary lymphoid organs. Since CXCL13 and LTα_1_β_2_ play key roles in TLO neogenesis, they might constitute potential biomarkers of TLO activity. The well-developed TLOs actively regulate local immune responses and influence disease progression, and they are thereby regarded as the powerhouses of local immunity. In this review, we recapitulated the determinants for TLOs development, with great emphasis on the fundamental role of chronic inflammation and tissue-resident stromal cells for TLO neogenesis, hence offering guidance for therapeutic interventions in TLO-associated diseases.

## Introduction

Inflammation is a process of physiological responses to pathological stimuli that involves immune cells and tissue-resident cells. The immune cells are mobilized to migrate to the infected or damaged tissues through the vasculature, and they release inflammatory mediators, such as cytokines and prostaglandins. These cells and inflammatory mediators are important players within the inflammatory microenvironment, although they differ at different phases of inflammation. At the early stage, the acute inflammatory response takes place within the first few hours due mainly to the infiltration of innate immune cells, including neutrophils, and is typically characterized by the appearance of redness, swollenness, and pain symptoms (1). At the later stage, the chronic cellular response involves the recruitment of not only innate immune cells but also adaptive immune cells. When antigens cannot be removed, the inflammation persists as chronic non-resolving inflammation in a site-specific manner. Such chronic inflammation serves as a common basis for immune cell-tissue resident stromal cell interactions and the accumulation of immune cells at sites of inflammation in a plethora of clinical diseases. The aggregates form segregated T-cell areas and B-cell follicles with follicular dendritic cells (FDCs) as well as neovascularization with newly formed blood vessels, lymph vessels, and high endothelial venules (HEVs) (2, 3). These structures have been described as tertiary lymphoid organs (TLOs), tertiary lymphoid structures, ectopic lymphoid organs, and lymphoid tissue neogenesis (4–6).

TLOs develop as ectopic lymphocyte clusters in connective tissues or parenchyma of diseased organs in various non-resolving chronic inflammatory diseases, including autoimmune diseases (7, 8), cancers (9), chronic infectious diseases (10), transplant rejections (11), and chronic inflammatory diseases like atherosclerosis (6, 12) and inflammatory bowel disease (13), indicating that chronic inflammation is the common promoter of TLO neogenesis. TLOs share remarkable cellular and structural similarities with secondary lymphoid organs (SLOs); for example, both contain various innate and adaptive immune cell types, T-cell areas, B-cell follicles, and HEVs (14). Both TLOs and SLOs require similar cellular and molecular developmental signals, albeit of their different source of origins. TLOs may play significant roles in local primary immune responses by mimicking SLO functions and, to some extent, can serve as protective or detrimental to organisms, though they are not encapsulated and can occur in any diseased tissues, unlike SLOs.

Cumulative evidence indicates that the ectopic expression of homeostatic lymphoid chemokines (i.e., CXCL13, CCL19, and CCL21) plays a vital role for TLO formation and contributes to leukocyte recruitment and their persistence in the tissues, while deficiency in any of these molecules can abrogate TLO formation. The resident stromal cells, including fibroblasts, endothelial cells, pericytes, and vascular smooth muscle cells (VSMCs), are the original producers of the chemokines that can functionally influence the ectopic infiltration of immune cells at the site of chronic inflammation (15, 16). Since TLOs harbor a number of immune cells, they may initiate local immune responses and ultimately influence the progression of diseases. In this review, we have summarized the current knowledge of TLOs—their definition, location, molecular determinants, and the potential roles they have in diseases—and discuss the functions of chronic inflammation and stromal cells in TLO formation.

## TLO Development

### The Definition of a TLO

**Figure 1:**
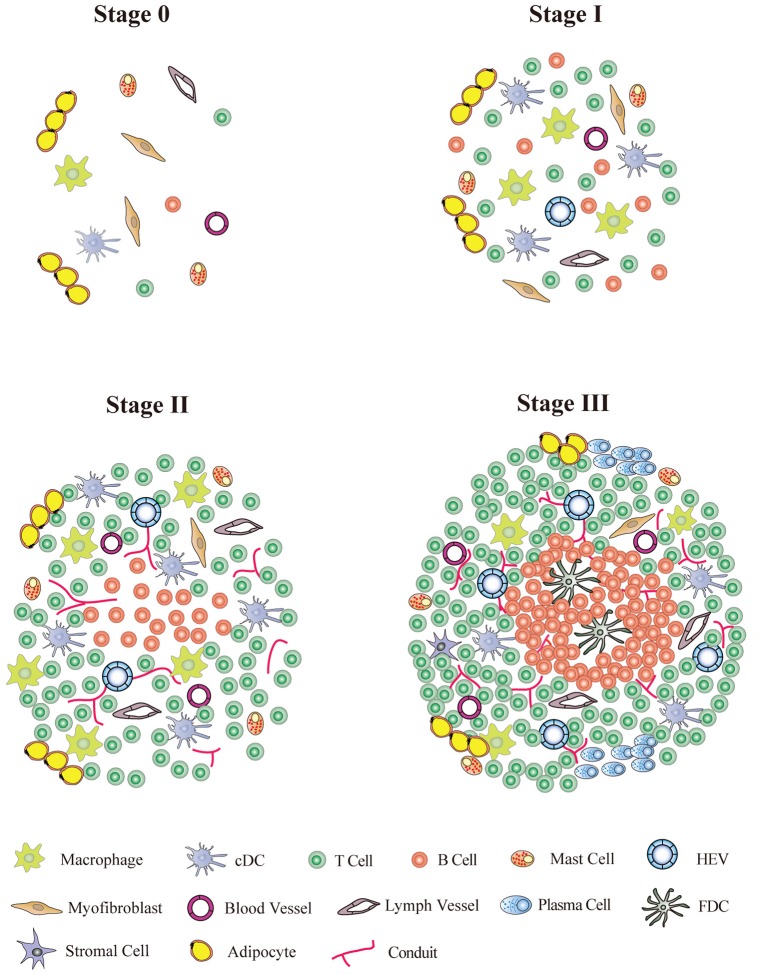
TLO stage classification. Stage 0, normal tissue without TLO formation; Stage I, early TLOs with mixed T/B-cell aggregates; Stage II, pre-TLOs with segregated T/B-cell area with lymph vessels and conduits; Stage III, well organized TLOs containing segregated T-cell area and B-cell follicles with germinal centers and follicle DC network.

TLOs are immune cell aggregates within or adjacent to the local tissues associated with chronic inflammation. TLOs share a structural homology with SLOs, including T-cell zones, B-cell follicles, stromal cells, HEV, blood, and lymphoid vessels. TLOs develop postnatally at undefined locations and are significantly associated with overwhelming antigen stimuli. SLO and primary lymphoid organ development, meanwhile, always occur in highly coordinated processes at predetermined locations during embryogenesis (17). The anatomy of TLOs is quite plastic, and the cellular composition varies from one model to the other. However, T/B cell segregation, vascular specialization, and certain lymphorganogenic chemokine expressions are the basic characteristics of TLOs; the well-developed TLOs with a germinal center (GC) and HEV formation are highly dependent on the disease stage and on the efficiency and duration of antigen-driven responses at the local site. Though TLOs have been extensively studied in the last 10 years, the standard definition of TLOs has not yet been set. Recently, Neyt et al. (18) proposed six criteria for determining TLOs: the presence of organized infiltrated T and B cells; the appearance of T-cell areas containing fibroblast reticular cell (FRC) networks; the presence of HEV; B cell class switching, GC reaction; activation-induced cytidine deaminase (AID) enzyme; and FDC presentation. However, it is not feasible to apply all of these criteria to each disease model nor is it possible to meet all criteria in different developing stages of the same disease. Based on the studies performed on artery TLO (ATLO) (13, 19, 20), we have suggested a definition of an ATLO based on three developmental stages: stage I has mixed T- and B-cell infiltrates; stage II contains segregated T- and B-cell areas with lymph vessels, HEVs, and conduits; and stage III has well-structured TLOs with segregated T- and B-cell follicles, activated GCs, and FDCs (Figure 1). However, these “criteria” are based on specific disease models and may be not applicable to other disease models as murine TLOs might differ from those in humans. Therefore, great effort is needed to set up the common criteria for defining TLOs for all disease models.

### The Spontaneous- and Induced- TLOs

**Figure 2:**
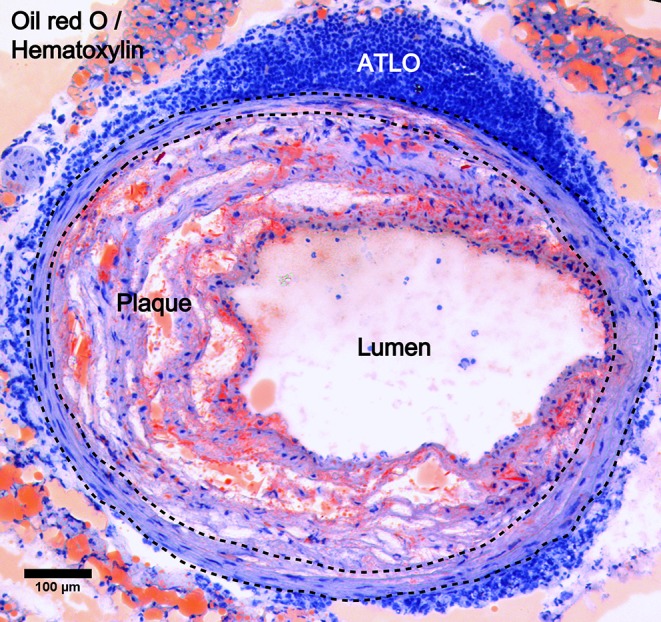
ATLO location in atherosclerotic aorta. Oil red O/Hematoxylin staining showing ATLO position in the abdominal aorta adventitia relative to media (dashed lines) and intimal plaque in aged *Apoe*^−/−^ mice.

TLOs can spontaneously develop in tissues with non-resolving chronic inflammation caused by persistent inflammatory stimuli or self-antigen during the disease progression. Their locations therefore vary depending on the disease models; tumor-associated TLOs, for example, are located peritumorally (21–23), intratumorally (24), or mixed (25). However, ATLOs are usually found in the adventitia of aged chow-fed *Apoe*^−/−^ mice (20) (Figure 2), which start to develop at around 55 weeks old post-birth and are fully formed at around 78 weeks old. Moreover, ATLO size correlates with the severity of intima atherosclerosis, and TLO-like structures were absent in the non-atherosclerotic arterial wall, thus indicating the importance of the local inflammation in the initiation and progression of ATLOs. Also, TLOs could develop in the central nervous system in autoimmune diseases, such as in the brain in multiple sclerosis (26). In the genetic non-obese diabetic (NOD) mouse model, TLOs arose in the pancreas with increased size and highly organized structure during disease progression (27). The locations of TLOs are shown in Table 1. In addition, TLOs can also be induced experimentally in some murine models. Administrating pathogens or inflammatory substances and the overexpression of lymphocyte-recruiting chemokines can result in the organoid assembly of lymphocytes (59). For example, significant lymphocytic infiltrates were detected in the thyroid gland of mice with CCL21 overexpression (60), and the inducible bronchus-associated lymphoid tissue (iBALT) developed in the lung of mice injected with a lipopolysaccharide (LPS) or influenza infection (51–53). The major difference between spontaneous and induced TLOs is that strong external stimuli are needed for the later for the induction of TLO formation, suggesting that there are difference in the intensities of immune responses. The immune activity in induced TLOs is variable, depending on the intensity of the stimuli applied to trigger them compared to spontaneous TLOs where immune activity might be low due to the prolonged chronic stimulation. However, the distinction between spontaneous and induced TLOs according to their immune activity and persistence is ambiguous since there is no standard to grade immune activity.

**Table 1:** Molecular determinant of TLOs in chronic diseases and experimental models.

**Diseases**	**Targeted organs**	**Species**	**Molecules/Signaling pathway/Cells**	**References**
**AUTOIMMUNE AND AUTOIMMUNE-RELATED DISEASES**				
Rheumatoid arthritis	Synovial tissue	Mouse	AID, LT, CCL21, IL-22, CCL19, CXCL12, CXCL13	(28, 29)
		Human	LT, IL-23, CCL21, CCL19, CXCL12, CXCL13	(29, 30)
Multiple sclerosis	Central nerve	Mouse	CXCL13, Podoplanin	(26)
	system	Human	CXCL12, CXCL13, AID	(31, 32)
Diabetes	Pancreatic islet	Mouse	CXCL13, CCL19, LT, TNFSF14-LTβR	(27, 33)
Sjogren's syndrome	Salivary glands	Mouse	IL-22, CXCL12, CXCL13	(5, 34)
		Human	CXCL13, CXCR5, CCR5	(35)
Myasthenia gravis	Thymus	Mouse	CXCL13	(36)
SLE	kidney	Human	CXCL13, CXCR5, BAFF, IL-21	(9, 12, 37)
Crohn's disease	Mesentery	Mouse	CCL19, CCL20, CCL21, CXCL13, CXCL16	(3)
Ulcerative colitis	Colon	Human	CCL21	(38)
**CHRONIC INFLAMMATION**				
Atherosclerosis	Aorta	Mouse	CXCL13, CCL21, CCL19, CCL20, LT, LTβR, ICAM1, VCAM1, MADCAM1, VEGFc	(14, 20)
		Human	CXCL13, CXCL16, CCL19, CCL20, CCL21, Tfh	(2, 39)
COPD	Lung	Mouse	CCL19, CXCL13, CXCL12, IL-17A	(40–42)
IBD	Gut	Mouse	CCL21, CCL19, CCR7	(8, 13)
		Human	CCL21, adipocyte	(3, 38)
**CANCERS**				
Breast cancer	Breast	Human	Tfh, CXCL13	(25, 43)
Lung cancer	Lung	Human	B cells, DCs	(44, 45)
Colorectal cancer	Colorectum	Human	AID	(46, 47)
Melanoma cancer	Skin	Human	CCL21, CCR7, AID	(48, 49)
**CHRONIC INFECTIOUS DISEASES**				
HCV	Liver	Human	CXCL13	(50)
Influenza virus	Lung	Mouse	CXCL12, CCL19, CXCL13	(51–53)
Helicobacter	Gastric mucosa	Mouse	CXCL13, CCR5, CCL21	(54)
Mycobacteriumtuberculosis	Lung	Mouse	CCL19, CXCL13, CCR7	(55)
**TRANSPLANTATION**				
Kidney failure	Kidney	Human	CXCL13, CCL19, CCL21	(56)
Cardiac failure	Heart	Mouse	CXCL13	(57, 58)

*SLE, Systemic lupus erythematosus; AID, activation-induced cytidine deaminase; LT, lymphotoxin; COPD, chronic obstructive pulmonary disease; SSA/Ro, Sjogren's syndrome antigen A (ribonucleoprotein autoantigen); SSB/La, Sjogren's syndrome antigen B (autoantigen La); IBD, inflammatory bowel disease; HCV, hepatitis C virus; ICAM1, intercellular adhesion molecule1; VCAM1, vascular cell adhesion molecule 1; MADCAM, mucosal addressin cell adhesion molecule 1; VEGF-C, vascular endothelial growth factor C*.

### Chronic Inflammation Favors Lymphoid Neogenesis

Inflammation is a self-limiting process, and multiple mechanisms ensure resolution in the normal condition. It is also a basic mediator of many diseases. TLOs are more frequently formed in chronic inflammatory diseases, although they are not present in all chronic inflammation. In contrast to SLO development, the inflammatory microenvironment provides the initial signal for TLO neogenesis, and chronic inflammation is sufficient to induce TLO formation even without the presence of lymphoid tissue inducer (LTi) cells (61), indicating that chronic inflammation is a fundamental player that favors lymphoid neogenesis.

Under chronic inflammatory conditions, resident stromal mesenchymal cells (62), such as fibroblasts, endothelial cells, VSMCs, and pericytes, are activated by proinflammatory molecules, including TNFα, LTα_1_β_2_, IL-17, IL-23, and chemokines, including CCL2, CCL3, CXCL9, CXCL10, and CXCL11 (1, 6, 63, 64). After activation, these cells are transformed into lymphoid tissue organizer (TLo)-like cells to release lymphorganogenic chemokines, such as CXCL13 and CCL21, to initiate the formation of TLOs (43, 62). For example, TNFα and LTα_1_β_2_, by interacting with TNFR1 and LTβR, respectively, activate medial VSMCs to acquire the phenotype of LTo cells. Most importantly, these activated VSMCs themselves produce high levels of CXCL13 and CCL21, thus promoting TLO formation in adventitia in atherosclerosis (14, 65). Moreover, the upregulated integrins on LTo or LTo-like cells, such as vascular cell adhesion molecule-1 and intercellular cell adhesion molecule-1, also facilitate the recruitment of immune cells to the local chronic inflammatory site, thus contributing to TLO formation (14, 15).

Locally or systemically, chronic inflammation persistently activates immune cells, upregulates LTα_1_β_2_ expression on their cell surfaces and releases cytokines, chemokines, or tissue factors, which act as indirect regulators of lymphoid organogenesis (66). Overexpression of TNFα by myeloid cells could induce intestinal TLOs in the absence of LTi cells (61). Similarly, IL-17A produced by Th17 cells alone overcomes the absence of LTi cells and mediates the formation of iBALT. The increased LTα-independent expression of CXCL13 was important for follicle formation during iBALT neogenesis (4). Both Th17 and LTi cells produce IL-22. Interestingly, *IL-22*^−/−^ mice showed a significant defect in early TLO formation and chemokine secretion because IL-22 is necessary for CXCL13 expression in fibroblast cells (5). In conclusion, regardless of the sources of the environmental mediators, the persistence of an inflammatory microenvironment is a clear factor that favors TLO formation.

### Roles of Innate and Adaptive Immune Cells in TLOs

Circulating monocyte-derived macrophages are known to migrate to certain tissues upon chronic inflammation, such as the salivary gland in Sjogren's syndrome (SS), and become a source of chemokines, such as CXCL13 and CXCL12, to promote TLO development (67). Unlike classical macrophages, tissue-resident macrophages are believed to be maintained by local proliferation and high expression of IL-7Rα (68), which is similar to LTi cells. It was reported that IL-7, together with CXCR5, promoted IL-7R upregulation in lymphoid organs (69). Moreover, overexpression of IL-7 resulted in TLO formation peripherally in a lymphotoxin (LT)α-dependent manner (70). It is possible that tissue-resident macrophages act as LTi cells and interact with IL-7-expressing stromal fibroblasts to initiate TLO formation. Moreover, TNF-expressing myeloid cells, such as macrophage and inflammatory monocytes, were required for fat-associated lymphoid clusters following in inflammatory challenge (71), and CD11c^+^ DCs were necessary for the maintenance of TLOs in the LPS-induced iBALT mouse model (72).

B cells are the predominant cell population during TLO neogenesis. In the later stage, advanced TLOs display features of B cell follicles comprising active GCs, indicating the proliferation and differentiation of reactive B cells. They could not only generate antibodies for immune responses, but also support local immune cell activation within the inflammatory tissues (6), and they play a critical role in both the initiation and organization of TLOs. B cells are important candidates for LT production, indicating that they might have a potential role in TLO neogenesis (73). Gene expression studies showed that B cells are recruited into TLOs by the interaction of chemokines CXCL13 and CXCL10 with their receptors CXCR5 and CXCR3, respectively (74, 75). Depletion of B cells from synovial tissue led to a reduction in T-cell derived IFNγ and IL-10 production, suggesting that synovial B cells control T-cell responses. In a chronic inflammatory mouse model, ATLOs harbor a high frequency of B cells which secrete large amounts of IgM and IgG antibodies. Although less numerous than B2 B cells, IL-10-expressing B1b cells present in ATLOs might exert key regulatory functions (76). Whether B1 B cells contribute to TLO maturation is not clear so far. The secreted antibodies by TLO B cells may have different effects on disease progression. These antibodies may serve as a double-edged sword, which could either have a positive role in infectious diseases or exacerbate autoimmune diseases. Since TLO B cells were also found in the synovium of inflamed joints, Rituximab (an anti-CD20 B-cell depleting antibody) might be an alternative therapy for treating rheumatoid arthritis, besides of TNF-α and IL-1 antagonist in clinic. It should know that Rituximab is an antibody for B-cell therapy in general, but not TLO specific. The role of B cells in the formation of TLOs in other autoimmune diseases, transplantation, and tumorigenesis events has been extensively reported (77). Therefore, the B cell aggregates could be regarded as the “marker” for TLO formation.

Regulatory T cells (Tregs) are believed to regulate the suppressive function in TLOs (6). In aged *Apoe*^−/−^ mice, both natural Tregs and induced Tregs were activated in ATLO during non-resolving chronic inflammation (20). Tregs in ATLOs may suppress the immune responses in plaque in a certain way because disruption of ATLOs by depletion of LTβR globally or specifically in VSMCs can abolish TLO formation and enhance atherosclerosis, suggesting that ATLOs have a protective role in disease progression (20). However, Clement M. et al. reported that regulatory CD8^+^ T cells controlled the development of TLOs and atherosclerosis, suggesting a pro-atherosclerotic role of TLOs in atherosclerosis (39). These conflicting conclusions from two individual studies might be due to some exterior factors, such as age, stage of disease, and environmental factors. Further studies are therefore warranted to investigate the precise role of TLOs in atherosclerosis.

### The Molecular Determinants of TLOs

The development of SLOs is a complex process that involves the interaction of LTα_1_β_2_-expressing hematopoietic LTi cells with LTβR-expressing non-hematopoietic stromal LTo cells. This interaction leads to the release of the cascade of adhesion molecules (e.g., intercellular adhesion molecule 1 and vascular cell adhesion molecule 1), cytokines (e.g., IFNγ, IL-17, IL-22, and IL-23) chemokines (e.g., CCL19, CCL21, CXCL12, and CXCL13), and the LTβR signaling pathway (18, 62). These secreted molecules and angiogenic growth factors regulate cellular and structural development of SLOs, while lymphangiogenic growth factors, like vascular endothelial growth factor C, induce lymphatic vessel development (78). Furthermore, LTo cells transdifferentiate into HEVs, FRCs, or FDCs, forming a network and facilitating T- and B-cell migration. The studies of TLOs in last 20 years have largely expanded our understanding of the molecular mechanisms involved in TLO formation. Though TLOs greatly resemble SLOs in cellularity and structure, the molecular determinants might be different in a certain way. Here, we discussed the vital role of TNFSF members, cytokines and chemokines that contribute TLO development.

### LT, LIGHT, and LTβR

The interaction of TNF superfamily members, i.e., LTα, LTβ, LIGHT (homologous to LT, also known as TNFSF14), and their receptor LTβR plays a critical role for lymphoid organ development. Over-expression of LTα alone or LTα and LTβ co-expression under rat insulin promoter II induced the development of LN-like structures with organized T- and B-cell areas and HEV in murine models (79), while LTα deficiency disorganized lymphocyte aggregation and HEV differentiation (51). In the NOD mouse model, LIGHT and LTβR expression were increased in the pancreatic TLOs (33), and the overexpression of LIGHT aggravated the disease (27). In addition, the overexpression of LTα or LIGHT induced by tumor cells resulted in intra-tumoral TLO development, indicating that the LTβR signaling pathway is crucial for the development of TLOs. Activation of LTβR in VSMCs was implicated in ATLO formation, whereas the interruption of the LTβR signaling pathway disturbed TLO formation with significantly reduced lymphocytic chemokine expression and HEVs development (20). Collectively, these data demonstrated that the LT-LTβR signaling pathway is crucial for the development, maintenance, and organization of TLOs.

### Cytokines

It is becoming increasingly clear that IL-17 is an important mediator for TLO development. The administration of LPS, viruses, or *M. tuberculosis* infection induced iBALT formation in an IL-17-dependent manner (80). IL-17 promotes inflammatory and homeostatic chemokine production, which is critical for iBALT initiation, while LT signaling is required for the differentiation of FRCs, FDC, and HEV formation in the later step of iBALT development. Moreover, IL-17^+^ CD4 T cells trigger TLO neogenesis in the central nervous system in the experimental autoimmune encephalitis model (26). TCRγδ T cells-derived IL-17 trigger stromal cells to release CXCL12 and thereby induce follicle formation in iBALT even in absence of FDCs (81). These data demonstrate that IL-17 is crucial for TLO formation.

Overexpression of IL-5 in the respiratory epithelium induced the formation of organized iBALT with epithelial hypertrophy, goblet cell hyperplasia, and accumulation of eosinophils in the airway lumen (82). IL-7, together with CXCR5, promotes TLO formation, and the overexpression of IL-7 thus resulted in TLO formation in non-lymphoid tissues (70). Furthermore, IL-27 inhibits TLO development and was proposed to be a novel therapeutic target in clinical treatment (83).

TNFα is known to promote the receptor expression of some inflammatory cytokines and is also proposed to engage with TLO development. It is likely that TNFα and induced proinflammatory cytokines convert resident stromal fibroblasts into functional LTo cells and initiate lymphoid neogenesis (84) because the ectopic expression of TNFα induces TLO formation in the periphery (63). It was further reported that IL-21, IL-22, and IL-17, produced by Th17, LTi, and γδT cells or neutrophils, are also important players in TLO formation. Increased IL-21 expression has been observed in TLOs in several disease models, such as RA and renal grafts (30, 83, 85). IL-22 promotes TLO development in salivary glands in response to local adenovirus infection (5), and TLO formation in human rheumatoid synovitis is strongly associated with the upregulation of IL-23, IL-21, IL-22, and IL-17F (30).

### Chemokines

Chemokines are known to influence leukocyte recruitment and TLO development. CXCL13 is predominantly expressed by fibroblastic stromal cells and regulates B-cell recruitment, differentiation, and maturation. Overexpression of CXCL13 by rat insulin promoter induced TLO formation characterized by T/B-cell zones and HEV (79). In advanced atherosclerosis, activated LTo-like VSMCs highly expressed CXCL13 and CCL21 to induce ATLO neogenesis. CXCL13, CCL21, and CXCL12, were also found in chronic inflammatory diseases, including SS, rheumatoid arthritis, and other disease models (Table 1). As a receptor for CXCL13, CXCR5 is of equal importance for TLO development since TLO has been shown to fail to develop in the absence of CXCR5 (86), indicating that individual chemokines or receptors have a significant impact on TLO development. Accumulating data demonstrated that CXCL13 and LTα_1_β_2_ might be the “bio-marker” predicting the formation of TLOs in some diseases, such as RA, SS, and atherosclerosis (87, 88).

CXCL12 is expressed by bone marrow stromal cells, SMCs, and HEVs in lymphoid organs. Transgenic mice with CXCL12 overexpression by RIP showed enriched infiltration of T and B cells, DCs, and plasma cells (89). Increased CXCL12 expression was detected in TLOs in the salivary glands of patients with SS (67). CCL19 and CCL21 are expressed by stromal cells and endothelial cells and interact with CCR7 to regulate T-cell homing during TLO neogenesis. Significant upregulation of CCL19 and CCL21 is observed in ectopic infiltrates of RA and SS (90), whereas CCL21 is more effective than CCL19 in forming TLOs (89). However, it was shown that CXCL12 alone could not promote TLO formation due to its inability to induce LTα_1_β_2_ expression (89), indicating that certain chemokines are not sufficient to drive the complete process of TLO formation alone. It might be that different chemokines have a differential capacity to recruit and maintain LTi cells and promote LTα_1_β_2_ expression, and they thus showed different abilities to promote TLO development (91).

Taken together, though many cytokines, chemokines, LTs, and receptors have been demonstrated for their roles in TLO development, it is inaccurate to claim that all of them could be used as the biomarkers for TLO formation because most of them perform the TLO-initiating function only in some specific models and local microenvironments. Nevertheless, CXCL13 and LTα_1_β_2_ could be candidate molecules that can be considered as potential biomarkers for TLO development.

### The Role of LTi Cells in TLO Development

LTi cells, one of two subgroups of type 3 innate lymphoid cells, are hematopoietic cells that were identified in fetal lymphoid tissues. They are critical for the induction of fetal lymphoid tissues, including LNs and Peyer's patches (92). LTi cells can be identified as CD45^+^CD3^−^CD4^+^ cells with a high expression of LTα_1_β_2_, RORγt, and IL-7Rα. One of the earliest events in secondary lymphoid organogenesis is the interaction between LTi cells and stromal LTo cells in the LN anlagen through LTβR signaling. Although LTi cells are known to be involved in the development of SLOs, the evidence for the requirement of LTi for TLO induction is still controversial as both LTi-dependent and independent TLO neogenesis have been reported.

Some evidence favors the contribution of LTi cells in the development of TLOs. Meier and colleagues reported that transgenic mice with IL-7 overexpression showed significantly increased amounts of LTi cells in the spleen and organized TLOs in the pancreas and salivary gland, suggesting that IL-7 stimulated LTi cells are essential for TLO development (70). In the CXCL13 transgenic model, the absence of LTi cells results in smaller and less organized cell aggregation, indicating the role of LTi cells in the development of TLOs (87). In human non-small cell lung cancer, tumor NCR^+^ ILC3 might contribute to tumor-associated TLOs by interacting with both tumor cells and tumor fibroblasts (93). Enhanced lymphocyte infiltrations in rheumatoid arthritis synovial fluid and murine intestine are believed to correlate with increased expression of TNF-related activation-induced cytokine by LTi-like cells (94, 95). Bone marrow derived M1 macrophages has been reported to act as LTi cells and crosstalk with VSMCs, and then initiated TLO formation in a LTβR-independent manner (96).

By contrast, some reports shed negative light on the role of LTi in TLO formation. Deletion of transcription factors, such as inhibitors of DNA-binding 2 (Id2) and ROR-γt, that are known to be critical for LTi-cell differentiation from lymphoid progenitors, lead to complete loss of LTi cells, LNs, and Peyer's patches but did not affect the spleen (97–99). However, when infected with influenza virus, the *Id2*^−/−^ or *Rorc*^−/−^ mice developed iBALT in the lung (10). Moreover, mice with overexpression of CCL21 in the thyroid developed ectopic lymphoid tissues, but, when they were crossed with *Id2*^−/−^ mice lacking LTi cells, the TLO formation was not interrupted (100). Importantly, Schropp et al. revealed that Th17 cells can act as substitutes for LTi cells and contribute to B-cell aggregate formation in the cerebellum in an experimental autoimmune encephalitis mouse model (101). These data indicated that LTi cells might be involved in but might not be essential for the formation of TLO. It is now gradually accepted that, during the chronic inflammatory condition, the TLO-inducing signals can be provided by cell types other than LTi cells (102) because lymphocytes, like B and T cells, are an alternative source of LT when appropriately stimulated (69).

### The Role of Stromal Fibroblast Cells in TLO Development

The adult stromal cells are derived from embryonic mesenchymal LTos and are considered to be structural components of an organ. They include fibroblasts, VSMCs, pericytes, epithelial cells, and blood and lymphatic endothelial cells (1). As traditionally considered, stromal cells not only make the architecture of the organ but also regulate the tissue function. Stromal fibroblasts are likely to act as scaffolds for the tissue due to their capacity to synthesize and remodel the extracellular matrix, and these cells in TLOs can be detected using specific marker combinations, such as gp38/CCL21 for FRCs and CD35/CXCL13 for FDCs. FRCs and FDCs in TLOs are predominantly associated with T- and B-cell recruitment, respectively. Therefore, fibroblasts closely interact with other cells to participate in tissue development, differentiation, and repair in the local microenvironment.

A key step in TLO formation is the “switching” of activated stromal cells toward an LTo-like phenotype. An increasing amount of evidence indicates that stromal cells are involved in orchestrating local immune responses and affecting disease progression (1, 63). During TLO neogenesis, tissue-resident stromal cells that gain the function of TLo-like fibroblasts represent a hallmark feature of TLOs due to the expression of lymph organogenic chemokines, such as CXCL13, CCL21, CXCL1, CXCL8, and CCL5 (1, 63). Adipose tissue-derived PDGFRβ^+^ stromal vascular cells have the capacity to differentiate into FDCs (103), while resident fibroblasts have been shown to massively proliferate and give rise to lymphoid stromal cells during inflammation and ontogeny (78). We previously proved that aortic VSMCs acted as LTo cells and highly expressed CXCL13, CCL21, and LTβR during long-term chronic inflammation in aged *Apoe*^−/−^ mice (16, 20). In humans, a gp38^+^ fibroblast showed phenotypical characteristics that were similar to FRC in SS patients, and CXCL13^+^ stromal cells were detected in synovial tissues of RA patients (38, 87). In addition, FAPα^+^ fibroblasts promoted inflammation and bone erosions in an arthritis mouse model (104), and Pdpn^+^ fibroblast were pivotal drivers of TLO formation independent of LT and RORγt (105). Guedj et al. found that mesenteric adipocytes orchestrated the development of functional TLOs in Crohn's disease-affected mesentery (3), suggesting that adipocytes may function as LTo-like cells and promote TLO formation. These data demonstrated that stromal fibroblast cells underwent a complex phenotypical change and acquired LTo-like characteristics for organizing local immune responses.

Emerging evidence indicates that stromal fibroblast cell differentiation is dependent on TNF family members, such as LTα and TNFα. LTα plays a significant role in lymphatic vessel function and in inflammation-associated lymphangiogenesis (106). *LT*α^−/−^ mice lacking LTα3 and LTα_1_β_2_ showed the absence of all LNs and PPs and the disrupted spleen architecture (107). *LTβ*^−/−^ mice, meanwhile, showed defects in LN development and spleen structure that were less pronounced than that in *LT*α^−/−^ mice (108). Furtado et al. showed that TNFα signaling pathways were sufficient to induce TLOs in the intestine independently of LTi cells, indicating that interactions between TNFα-expressing myeloid cells and stromal cells were enough to induce TLOs formation (61). IL-17A induced iBALT formation in the lung by directly stimulating the differentiation of CXCL13- and CCL21-expressing stromal cells (51). Furthermore, IL-23 and IL-22 are known to activate fibroblasts from the lung and salivary gland, respectively, to express CXCL13 for TLO formation (5, 80). These data indicated that TNF, LT, and some leukocytes, including myeloid cells and granulocytes that release pro-inflammatory cytokine, are capable of activating resident fibroblasts for their transdifferentiation (5).

## TLOs in Diseases

The potential impacts of TLOs on disease progression are increasingly gaining recognition. Autoimmune diseases are abnormal conditions with both B- and T-cell responses against self-tissue antigens. TLOs have been witnessed in various autoimmune diseases, especially in the affected areas, like joints of rheumatoid arthritis (28), meninges in multiple sclerosis (109, 110), salivary glands in SS (111), pancreas in diabetes (112), and the thyroid in Hashimoto's thyroiditis (113) (Table 1). The formation of TLOs in autoimmune diseases may contribute to disease manifestation.

TLOs with activated GCs also expressed the enzyme AID, which mediates B-cell clonal expansion and somatic hypermutation of the V(H) gene within TLOs. Plasma cells then release high-affinity antibodies, such as auto-antibodies targeting ribonucleo proteins Ro and La (Sjogren's syndrome antigen A and Sjogren's syndrome antigen B, respectively) in SS (76). During this process, generated autoreactive B cells in TLO GCs could escape negative selection and apoptosis. For instance, GC B cells from TLOs of SS demonstrate increased anti-apoptotic BCL-2 expression and decreased rate of apoptosis (114). These data demonstrate that TLO GCs provide the microenvironment for antigen-specific antibody production. However, the GCs and accumulated disease-specific autoantibodies could induce adaptive immune responses in local tissues, which perpetuate autoimmune diseases. Therefore, targeting surface antigens on B cells has emerged as a promising therapeutic approach. For example, proteasome inhibitors and antibodies against CD138, B-cell maturation antigen (BCMA), and the Signaling Lymphocyte Activation Molecule family (SLAMF) were used in clinical trials (16, 17, 115). Though limited efficiency in clinical practice is seen, a combination of different therapies could bring about improvement in practical terms. In addition, rituximab (a CD20-depleting antibody that targets B cells before the stage of plasma blast cells) may also be a better alternative for treating rheumatoid arthritis.

TLOs also harbor T follicular helper (Tfh) cells that are critical in T-cell dependent B-cell responses and therefore regulate active GC responses in the lymphoid organ. The interaction of ICOS, CD40, and IL-21 on Tfh cells with ICOSL, CD40L, and IL-21R expressed on GC B cells induces the activation, affinity maturation of B cells, and further differentiation of plasma cells in TLOs (77). It was also reported that Tfh-GC B-cell axis was proatherogenic in an *Apoe*^−/−^ mouse model (39). Therefore, blocking B-Tfh cell interaction through targeting ICOS-ICOSL, CD40-CD40L, or IL-21-IL-21R could significantly affect downstream TLO B-cell activation.

Besides autoimmune diseases, TLOs also develop in some infectious diseases and various cancers (Table 1), and they are generally believed to perform a protective role by skewing the local immune responses toward anti-infection and anti-tumor outcomes. However, the efficiency of the immune responses may change according to the local concentration of viral and tumor antigens. In a long-lasting inflammation, the synthesis of cytokines, chemokines, and tissue factors is constantly enhanced. Ectopically developed TLOs will mount the local immune responses and deteriorate the disease progression. Therefore, the roles of TLOs in diseases are largely dependent on the local conditions, either protective or harmful.

## Clinical Implications

TLO formation may affect the disease process because ectopic GCs in TLOs could potentiate local immune responses. As discussed above, TLOs might be protective in certain diseases, such as infectious diseases, cancers, and atherosclerosis, because they can mount an efficient response against antigenic stimuli in tumors or chronic inflammation. However, in other diseases, including autoimmune diseases and transplantation, TLOs might further aggravate disease progression (62, 66). Therefore, promoting or inhibiting TLO development with tailored immune therapy should be decided based on the disease phenotype.

Given the prominent role of LT in TLO development, targeting the LTα_1_β_2_-LTβR signaling pathway has already been used extensively to modulate TLO formation. The decoy receptor–LTβR-Ig fusion protein is used widely to interfere with the signaling pathway in the animal model and preclinical trials (116). It should be noted that LTβR-Ig treatment disturbed spleen microarchitecture, including loss of integrity of marginal zones, FDCs, and cell proliferation (14), and this is suggestive of its side effect on SLOs. The administration of LTβR-Ig prevents insulitis at the early stage, reverses insulitis, and disrupts pancreatic lymphoid aggregates at the late stage of the disease in NOD mice (117). In a mouse model of autoimmune sialoadenitis, LTβR-Ig treatment resulted in decreased immune cell infiltration and a significant improvement in the salivary gland function (118). Notably, vascular smooth muscle cell-specific deletion of LTβR in atherosclerotic mice disrupted adventitial TLO formation and aggravated the atherosclerotic plaque size (20). Thus, the detailed mechanism of disease progression still require further investigation.

Importantly, pateclizumab (a monoclonal antibody against LTα) and baminercept (LTβR-IgG1, an inhibitor of both the LTα_1_β_2_ and LIGHT pathways) are currently undergoing Phase I and II trials for the treatment of autoimmune diseases (119). Pateclizumab, a humanized monoclonal antibody against LTα, is already verified to be generally safe and well tolerated in clinical trials (119, 120). Unexpectedly, 6 months of baminercept treatment failed to improve glandular dysfunction in patients with primary SS. Although not achieving expected treatment efficacy, the studies revealed, to some extent, the therapeutic effect of baminercept in LT/LIGHT-dependent pathways, suggesting that blocking LTβR signaling might be of therapeutic benefit at earlier stages (121). Both treatments resulted in decreased serum CXCL13 levels in patients with RA, but baminercept treatment was unable to diminish the serum levels of BAFF, LIGHT, or IP-10 compared to the placebo group (119, 120).

The blockade of a TLO-associated chemokine or its receptor is another therapeutic strategy that has been tested in animal models. Chemokine CXCL13, CCL19, and CCL21 and the receptors CXCR5 and CCR7 play central roles in lymphocyte migration during TLO formation. Targeting these molecules might therefore be pivotal for the therapeutic inhibition of TLOs. It has been reported that the blockade of CXCL13 in mouse models reduces glandular inflammation in SS (122) and decreases the severity of collagen-induced arthritis and GC formation in synovial tissues (123). Despite this, TLO-associated chemokine/receptor-based treatments have not yet entered clinical trials.

Furthermore, other therapeutic strategies targeting TLO-associated molecules or pathways have been launched recently, including Tfh-related molecules, i.e., IL-21 and the receptor IL-21R, co-stimulatory molecules ICOS and its ligand ICOSL, and the IL-17 pathway (66). The pharmacological blockade of these targets has been shown to have a potential function in the experimental disease models, some of which have already entered clinical trials (124).

## Concluding Remarks

Chronic inflammation promotes neogenesis of TLOs to control local immunity in a feedback manner. The potential impact of TLOs on disease progression, either protective or damaging, is increasingly attracting peoples' attention. Although considerable progress has been made in the understanding of ectopically developed lymphoid organs, many basic questions remain unsolved. Since TLOs are different from simple leukocyte infiltrates in affected tissues of chronic diseases, a globalized standard for defining and identifying TLOs could be fundamental for all future studies. In the meantime, TLOs can serve as a potential therapeutic target in clinical practices where early diagnosis of TLOs and corresponding appropriate therapies are promising. Nevertheless, specific diagnostic approaches, or biomarkers, to predict TLO development at the early stage of disease are still lacking. Extensive studies on cellular and molecular mechanisms of TLO development are also still required. With regards to therapies, all these aspects should be considered before applying systemic drugs to modulate TLO formation as they could have serious side effects on other lymphoid and non-lymphoid tissue compartments.

## Author Contributions

All authors listed have made a substantial, direct and intellectual contribution to the work, and approved it for publication.

### Conflict of Interest

The authors declare that the research was conducted in the absence of any commercial or financial relationships that could be construed as a potential conflict of interest.
